# Integrated electrocoagulation-flotation of microalgae to produce Mg-laden microalgal biochar for seeding struvite crystallization

**DOI:** 10.1038/s41598-022-15527-6

**Published:** 2022-07-06

**Authors:** Krishnamoorthy Nageshwari, Scott X. Chang, Paramasivan Balasubramanian

**Affiliations:** 1 Department of Biotechnology and Medical Engineering, National Institute of Technology Rourkela, Rourkela, 769008 Odisha India; 2 Department of Renewable Resources, University of Alberta, Edmonton, AB T6G 2E3 Canada

**Keywords:** Environmental biotechnology, Environmental chemistry

## Abstract

Developing sustainable materials for recovering and recycling nutrients from wastewater is critically needed for nutrients such as phosphorus that have a diminishing supply. Struvite crystallization is emerging as a promising strategy for phosphorus recovery which can be enhanced with seeding through microalgal biochar. The main bottleneck of using microalgae is its high harvesting cost. In this study, an integrated electrocoagulation-flotation (ECF) process is used to recover and at the same time modify the algal surface with magnesium anode and inert carbon cathode. Harvesting efficiency of 98% was achieved with 40.78 mA cm^−2^, 0.5 cm inter-electrode distance and energy consumption of 4.03 kWh kg^−1^ in 15 min. The harvested microalgae were pyrolyzed to obtain a yield of 52.90% Mg-laden microalgal biochar. Simultaneously, surface impregnation of 28% magnesium was attained as confirmed by Scanning electron microscopy (SEM) and energy dispersive X-ray spectroscopy (EDS). Phosphorus recovery and struvite yield of 93.70% and 2.66 g L^−1^, respectively, were obtained from dosing 1.50 g L^−1^ Mg-laden microalgal biochar. Comparison of physicochemical characteristics of residual supernatant after microalgal harvesting and struvite recovery showed that the combined use of both the residuals can serve as a sustainable growth medium for microalgae. The overall operating cost of the integrated process was found to be 2.48 USD kg^−1^ with a total energy consumption of 10.76 kWh kg^−1^, which was found to be lower than conventional harvesting unit processes such as centrifugation and filtration. This novel approach can help attaining a circular bioeconomy by encompassing nutrient recovery and waste management in an integrated process.

## Introduction

The alarming depletion of phosphorus (P) rock resource has triggered a rising need for the recycling and recovery of P, a non-renewable resource. The world’s P rock reserve is predicted to be depleted in the next 100 years. Retrieval of P from nutrient-rich wastewaters in the form of struvite can be a potential solution for cessation of the leaky P cycle. In addition, this strategy can prevent eutrophication and algal blooms in natural water reservoirs. This outlook has gained spotlight in recent years due to the following advantages: struvite is a slow-release fertilizer, the impurities present in the recovered struvite are very low and all major essential nutrients required by plants are present^1–3^. Though the approach of struvite recovery leads to a win–win strategy for both the environment and the society, the efficiency of struvite recovery critically depends on the size of the crystals formed. Most of the struvite crystals are fine-sized and are undesirably lost during retrieval^4^.

Seeding is an alternative strategy to enhance nucleation by providing enormous active sites for crystal growth in a relatively short induction time. Biochar, a carbonaceous substance produced from pyrolysis, synthesized from several biomasses are being employed as a seeding material to improve struvite precipitation. Biochar has been proven to possess properties such as high surface area, porosity and cation exchange capacity, and has been applied for nutrient adsorption from wastewater and as a soil amendment^5–7^. In this context, production of biochar from microalgae is emerging as a new technology for sustainable biochar production due to the high nutrient content of P, nitrogen and other inorganic elements that have agronomic importance. Additionally, microalgae can fix carbon and mitigate greenhouse gas emissions^32,40^. Microalgal biochars produced at higher pyrolysis temperatures possess high micropore volume and O-containing functional groups that can effectively serve the purpose of seeding crystal nucleation^8^. These advantages can be exploited for seeding struvite crystallization to minimize the induction time and enhance phosphate recovery and crystal size. However, due to the small cell size, low cell concentration and culture density of microalgae as equal to water, the cost and energy associated with harvesting microalgae makes their utilization uneconomical^41,42^. Among the existing harvesting techniques, coagulation, flocculation, filtration, and centrifugation are found to be more appropriate for practical applications. These techniques are sometimes employed in combination to further enhance the efficiency of the process^43^.

In recent years, electrochemical techniques are developing as biomass surface modification strategies prior to pyrolysis, serving the purposes of ion impregnation and surface area enhancement for efficient adsorption of nutrients and heavy metals. Chabi et al*.*^9^ modified microalgae *Chlorella sp.* (PTCC 6010) by subjecting to electrocoagulation, in an electrolysis system containing aluminium-boron electrodes for adsorbing tetracycline. Similarly, Jung et al*.*^10^ fabricated biochar by electrically pre-treating *Laminaria japonica* with aluminium electrode for adsorption of phosphate. These electrochemical techniques can save time, reduce complexity and processing cost and improve efficacy.

Magnesium is relatively less toxic than aluminium or iron; however, the studies related to use of magnesium for harvesting or electro-modification is very scarce^11^. The potential of magnesium for the above-said applications and the mechanism behind should be explored further. In addition, research on microalgal biochar is just gaining interest and while the previous studies have focussed on application of the modified biochar as an adsorbent for nutrient recovery or pollutant removal from wastewater, its role as a seeding material for struvite crystallization has not been explored yet. Such a direction of combining struvite and microalgal biochar can benefit production of a ready-to-use, slow-release organic fertilizer consisting of all the nutrients required for crop cultivation. Also, the best use of the residual liquid after microalgal harvesting and struvite precipitation needs to be researched. This manuscript aims to address these research gaps by economically impregnating the microalgal biomass with magnesium through electrocoagulation-flotation harvesting technique and utilization of the intended biochar not only as a seed but also a source of external source of magnesium for struvite crystallization. The residual supernatant can be used for subsequent microalgal cultivation to reduce the overall water footprint of the process. This integrated system not only uses the materials consecutively generated from each step of the process but also minimizes the cost and reuses the waste produced. Such utilization of microalgae for material synthesis alongside nutrient recovery from wastewater contributes to the regenerative economy along with sustainable production and consumption of resources through value-added products. These strategies can help reduce excess build-up of regional by-products and hence gain significance on national and international levels. Hence, this work highlights the benefits of the integrated process in wastewater remediation, CO_2_ mitigation and consecutive cultivation and usage of the microalgal biomass to stimulate circular economy and sustainability. To the best of the author’s knowledge, this is the foremost study conducted on utilizing engineered microalgal biochar as a seed material for struvite crystallization. The interplay between the resource recovery and wastewater management will create a new research field of vast importance and further exploration of concepts.

## Results

### Microalgal cultivation

In this study, the highest microalgal concentration of 13.72 g wet weight L^−1^ or 1.96 g dry weight L^−1^ was achieved when cultivated for a period of 25 days. At this stage, the microalgal growth was observed to shift from exponential phase to stationary phase as suitable for harvesting.

### Optimization of parameters for electrocoagulation-flotation harvesting of microalgae

In this study, magnesium sacrificial anode was utilized not only to harvest microalgae but also to impregnate Mg^2+^ ions on the surface of microalgal cells as a pre-treatment strategy. The mechanisms taking place during this process and the ECF set-up used in the experiments are shown in Supplementary Fig. S1. The medium was initially stirred at higher rpm and then lower rpm to enhance dispersion of ions, floc formation, avoid settling of bigger flocs and assist flotation.

**Figure 1:**
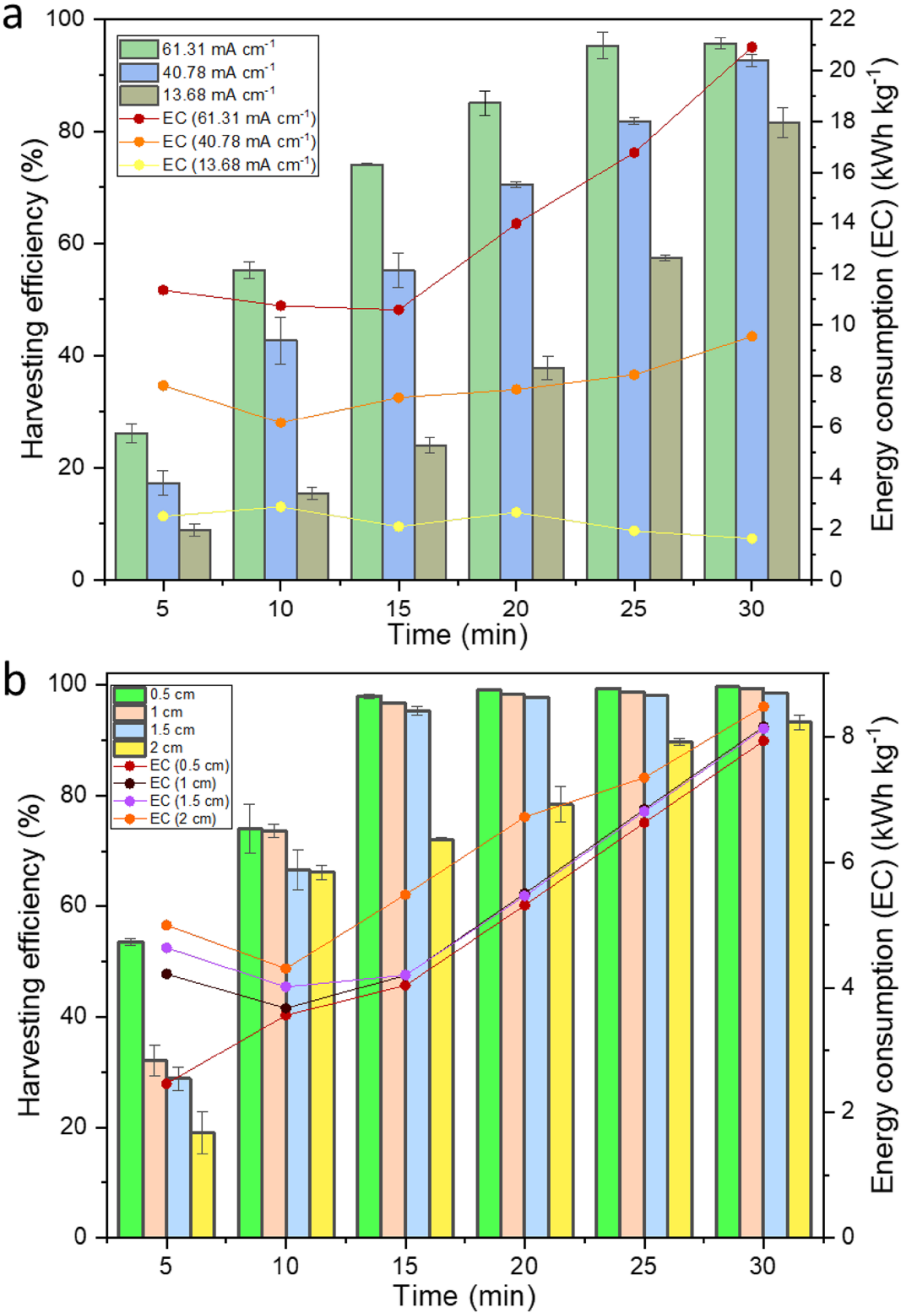
Optimization of (**a**) current density and (**b**) interelectrode distance for energy-efficient microalgal recovery with respect to time.

A combined galvanostatic and potentiostatic approach was followed where voltage was varied in the DC power supply unit to change the current input required for microalgal harvesting. Three different current densities (CD) (61.31 mA cm^−2^ (30 V), 40.78 mA cm^−2^ (20 V) and 13.68 mA cm^−2^ (10 V) were supplied for a period of 30 min with fixed interelectrode distance of 1 cm and pH of 8.0. It can be observed from Fig. 1a that harvesting efficiency increases with increase in CD and highest (95.6 ± 0.98%) for 61.31 mA cm^−2^ at 30 min. The energy required to attain at least 80% harvesting efficiency is 13.97 kWh kg^−1^ (20 min), 8.05 kWh kg^−1^ (25 min) and 1.62 kWh kg^−1^ (30 min) for 61.31, 40.78 and 13.68 mA cm^−2^, respectively (Fig. 1a). In other words, the energy consumed is ~ 10 times more for 61.31 mA cm^−2^ than for 13.68 mA cm^−2^, with a time difference of 10 min. In fact, an optimum CD is crucial for striking a balance between recovery efficiency, energy consumption (EC) and operational costs. Though from an economic point of view, CD of 13.68 mA cm^−2^ could be optimum, considering the integrity of microalgal cells and its components, 40.78 mA cm^−2^ was considered as the optimum CD for microalgal recovery in further experiments.

Interelectrode distance (IED) also has a key role in microalgal harvesting and energy consumption. In this study, IED was optimized by varying the distance from 0.5 to 2 cm, with constant supply of CD (40.78 mA cm^−2^) for 30 min. It can be seen in Fig. 1b that recovery efficiency is higher in case of shorter electrode distance and vice versa. The efficiency reached almost 53.5 ± 0.64% in the first 5 min and escalated up to 98.0 ± 0.40% in 15 min for 0.5 cm IED. After 15 min, the recovery efficiency stabilized for all IED irrespective of time.

The results of the optimization experiments conducted in this work show that an IED of 0.5 cm and CD supply of 40.78 mA cm^−2^ for 15 min would be ideal to obtain microalgal harvesting efficiency of 98.0 ± 0.40% with EC of 4.03 kWh kg^−1^.

### Study of variation in physicochemical characteristics of algal medium before and after harvesting

pH determines the rate of hydroxyl ion release and speciation of metal ions in the medium and in turn influences the microalgal removal kinetics at every stage of floc formation. In this study, the initial pH was 9.7, which increased gradually during the harvesting process and reached 10.7 at the end (Supplementary Fig. S2a). The electrical conductivity and salinity increased from 809 to 823 µS cm^−1^ and 0.4 to 0.42 psu, respectively, as a result of Mg^2+^ and OH^−^ ion release in the medium (Supplementary Fig. S2b,d). The total dissolved solids (TDS) of the solution were observed to increase from 401 to 412 ppm (Supplementary Fig. S2c). The difference in the conductivity and salinity, before and after harvesting, is much lower because no salts were added into the media. Once the ion concentration released from the anode was sufficient enough to flocculate all the cells, the concentration in the effluent remained minimum. Similarly, the Mg^2+^ ion concentration in the residual supernatant after ECF was very low (< 2 mg L^−1^). The conductivity and salinity were sufficient enough to obtain 98% harvesting efficiency in 15 min with an EC of 4.03 kWh kg^−1^, which is lower than energy consumed by other harvesting techniques (Supplementary Table S1).

### Pyrolysis of pre-treated microalgae and comparison of characteristics between chemical and electro-modified biochar

**Figure 2:**
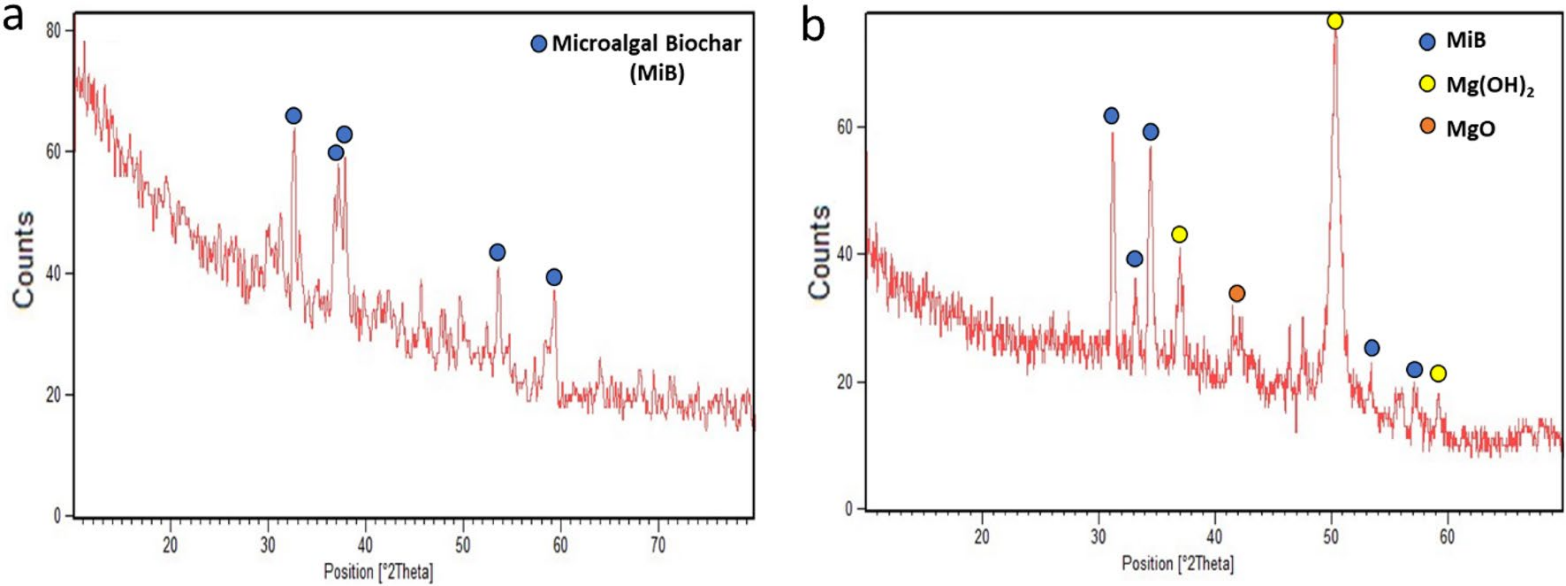
X-Ray diffraction graphs of (**a**) microalgal biochar^12^ (**b**) Mg-laden microalgal biochar used in this study.

The harvested microalgal cells were dried and pyrolyzed at 480 °C under oxygen limited conditions. A yield of 52.90% Mg-MiB was obtained from the pre-treated algal biomass. The effect of electro-modification and magnesium impregnation on the surface of biochar was studied using XRD analysis. New peaks of Mg(OH)_2_ and MgO were clearly observed on the modified-biochar (Fig. 2). The diffraction peak at 42.3° was identified as MgO (JCPDS 75-0447) and the peaks at 37.1, 50.4 and 59.2° were found to be Mg(OH)_2_ (JCPDS 00-007-0239). The high intensity of peaks confirmed that Mg(OH)_2_ is the main crystalline phase and were uniformly incorporated throughout the surface of Mg-MiB. This means that magnesium on the surface will be available as Mg^2+^ for spontaneous struvite formation. The other significant peaks at 31.16, 33.16, 34.4, 53.5 and 57.4° indicate carbon oxides and graphite planes of microalgal biochar (JCPDS No. 76-2378).

**Figure 3:**
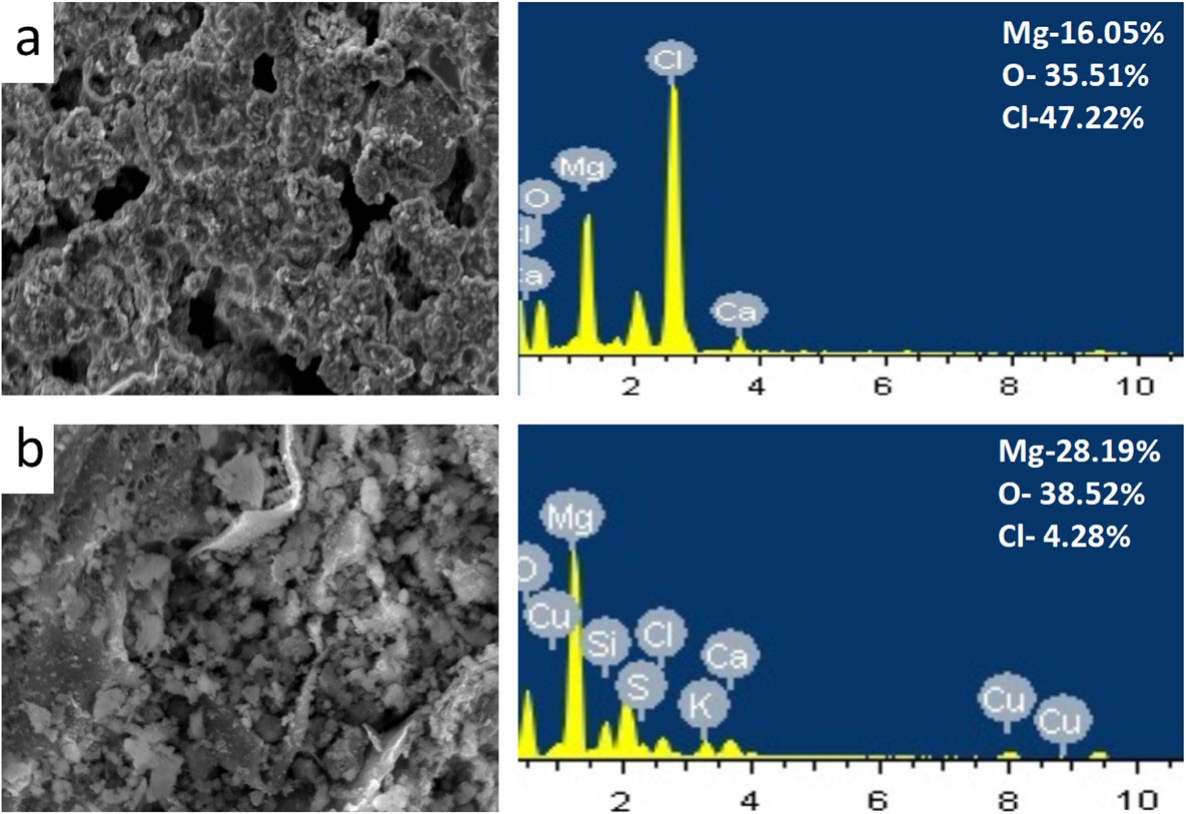
Scanning electron microscopy-energy dispersive X-ray spectroscopy analysis of (**a**) chemical-modified and (**b**) electro-modified Mg-laden microalgal biochar.

SEM analysis revealed the structural variations incurred with magnesium pre-treatment. The biochar can be seen to have a large surface area and microporous structure with average pore diameter of electro-modified Mg-MiB was 2 µm, which acts as an active site for the deposition of crystals (Supplementary Fig. S3). The rough and craggy surfaces of the modified biochar indicate the presence of nano-structured Mg(OH)_2_ which are the chief adsorption spots for struvite crystallization. The magnesium content of the biochar was studied with EDS. In addition, the effectiveness of the impregnation with ECF was compared with chemically modified microalgal biochar in this study. It was observed that the magnesium concentration was approximately 28 times and 16 times higher in case of electro-modified and chemical-modified biochar, respectively, with respect to the control (Fig. 3). In spite of the inherent oxygen content of microalgal biochar (Fig. S5)), formation of O-containing groups on the surface contributed to the overall oxygen concentration which was higher in electro-modification (4.22%) than chemical modification (1.21%).

### Struvite crystallization using Mg-laden microalgal biochar seeds and characterization

To obtain maximum struvite recovery with minimum resource utilization, optimization of Mg-MiB dosage was carried out. The dosage was varied between 0.50 and 3.00 g L^−1^ and all other process parameters were maintained the same for all experiments. It can be observed from Table 1 that the PO_4_^3−^ recovery increases with increase in Mg-MiB dosage. However, there was no significant trend or difference in case of NH_4_^+^ recovery. Once the Mg^2+^ ion leached in the medium was sufficient to react with PO_4_^3−^ and NH_4_^+^ ions, the residual concentration increased with dosage. The presence of higher residual Mg^2+^ in the synthetic medium means that excess magnesium has been supplied, which is unnecessary considering lack of PO_4_^3−^ to react and spending of fresh Mg-MiB. The struvite yield also increased with dosage until 1.50 g Mg-MiB L^−1^ followed by a slight decrease. Taking both input resources (dose, energy, and cost) and outcome variables (phosphate and ammonium recovery, residual Mg^2+^ concentration and struvite yield) into consideration, 1.50 g L^−1^ was be concluded as the optimum Mg-MiB dosage for attaining PO_4_^3−^ recovery of 93.72% and highest struvite yield of 2.66 g L^−1^. In this case, the NH_4_^+^ recovery was greater than 95% and the Mg^2+^ concentration in residual supernatant was lower.

**Table 1:** Optimization of Mg-laden biochar dosage for enhanced PO_4_^3−^, and NH_4_^+^ recovery and struvite yield.

Mg-laden biochar dosage (g L^−1^)	PO_4_^3−^ recovery (%)	NH_4_^+^ recovery (%)	Residual Mg^2+^ (mg L^−1^)	Struvite yield (g L^−1^)
0.50	72.0 ± 0.06	96.6 ± 0.13	15.0 ± 1.02	1.58 ± 0.08
1.00	90.5 ± 0.03	96.3 ± 0.06	52.5 ± 2.04	2.55 ± 0.02
1.50	93.7 ± 0.10	95.9 ± 0.09	62.5 ± 2.06	2.66 ± 0.01
2.00	95.9 ± 0.007	95.3 ± 0.04	75.6 ± 5.61	2.43 ± 0.06
2.50	96.2 ± 0.009	94.6 ± 0.09	108.8 ± 3.06	2.30 ± 0.06
3.00	99.1 ± 0.19	94.2 ± 0.03	190.6 ± 2.55	2.52 ± 0.05

For positive control, MgCl_2_.6H_2_O equivalent to the magnesium loaded in 1.50 g Mg-MiB L^−1^ was used to precipitate synthetic struvite for comparison of nutrient recovery, struvite yield and quality. The results revealed that chemical supplementation of magnesium yielded 82.0 ± 0.68% and 75.0 ± 0.71% of PO_4_^3−^ and NH_4_^+^ recovery, respectively, with a struvite yield of 1.12 ± 0.29 g L^−1^. In this case, the residual magnesium concentration was very high (627 ± 9.22 mg L^−1^).

### Qualitative analysis of struvite-microalgal biochar composite

**Figure 4:**
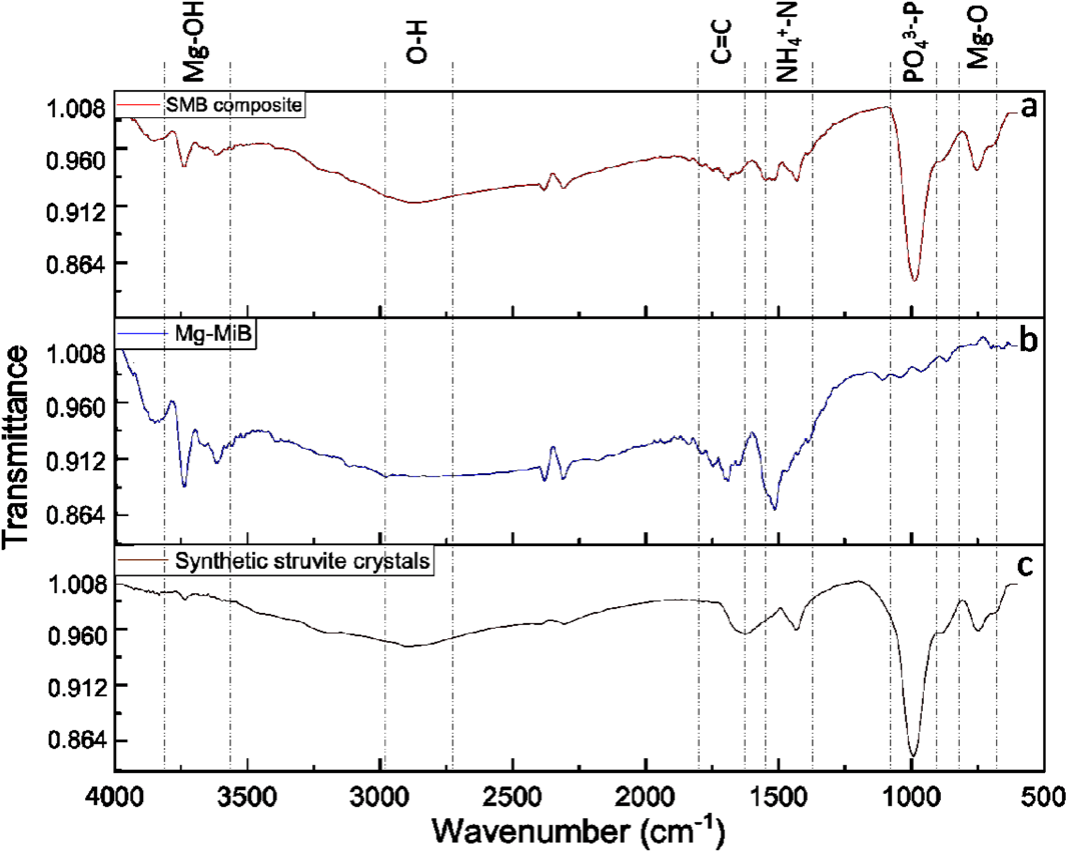
Fourier transform infrared spectroscopy graphs of (**a**) struvite-microalgal biochar composite. (**b**) Mg-laden microalgal biochar. (**c**) synthetic struvite crystals.

Particle size analysis revealed the average size of Mg-MiB and SMB composite to be 5.538 and 8.471 mm in diameter. The size of Mg-MiB grew with deposition of struvite crystals on its surface. Structural variation was also observed between struvite produced using MgCl_2_.6H_2_O and Mg-MiB using SEM analysis. FTIR analysis of Mg-MiB confirms that the impregnated magnesium is present as Mg(OH)_2_ on the surface of microalgal biochar (stretching vibrations at 3614, 3740 cm^−1^) (Fig. 4). The peak at 1687 cm^−1^ indicates C=C aromatic rings in the pyrolyzed biomass. Due to the adsorption of struvite on Mg-MiB, characteristics peaks of PO_4_^3−^ and NH_4_^+^ were observed at 993 and 1430 cm^−1^. The asymmetric range of bands between 2800 and 3500 cm^−1^ represents –OH and –(OH)_2_ due to adsorption of water and other substituted groups^13^. Peaks at 1669 and 1697 cm^−1^ correspond to N–H amide bonds and C=O stretching of SMB composite, respectively. It is noteworthy to mention the shift in Mg-OH to Mg-O upon formation of adsorption of struvite which can be due to new bonds established between Mg-O and PO_4_^3−^ (Mg-O-P)^14^.

### Utilization of residual supernatants after harvesting and struvite precipitation for microalgal cultivation

The physicochemical parameters and nutrient composition of the residual supernatants used for the analysis are listed in Table 2. The pH of supernatant after microalgal harvesting and struvite recovery was measured to be 10.73 and 8.8, respectively. Other characteristics such as EC, salinity and TDS are slightly lower and higher (not insufficient or intolerable) in microalgal and struvite supernatants, respectively, compared to the laboratory optimized microalgal culture media requirements (6.5% v/v urine). Comparing the concentration of essential nutrients, phosphate is lower than the required level (~ 10 times), while ammonium concentration should be sufficient enough. Similarly, nitrate, calcium and potassium match the nutrient prerequisite for algal cultivation. In case of magnesium and sodium, the concentration in residual supernatant after struvite recovery is higher due to the residual Mg^2+^ from Mg-MiB and salts of sodium used for the preparation of synthetic urine, respectively.

**Table 2:** Comparison of physicochemical characteristics of microalgal cultivation media and residual supernatants after harvesting and struvite recovery.

Physicochemical characteristics	Microalgal cultivation media	Residual supernatant after microalgal harvesting	Residual supernatant after struvite recovery
pH	8.63 ± 0.04	10.70 ± 0.04	8.81 ± 0.08
Electrical conductivity (mS cm^−1^)	1.99 ± 0.002	0.82 ± 0.001	12.70 ± 0.016
Salinity (psu)	1.01 ± 0.004	0.42 ± 0.008	7.29 ± 0.024
Total dissolved solids (ppm)	997.00 ± 3.00	412.00 ± 1.00	363.00 ± 2.00
Phosphate (mg L^−1^)	133.33 ± 19.99	1.27 ± 0.09	10.34 ± 0.19
Ammonium (mg L^−1^)	25.18 ± 1.72	4.13 ± 0.63	33.25 ± 0.08
Nitrate (mg L^−1^)	53.70 ± 6.21	16.70 ± 0.21	35.40 ± 0.36
Magnesium (mg L^−1^)	4.58 ± 1.17	1.66 ± 0.58	62.50 ± 3.06
Calcium (mg L^−1^)	7.96 ± 0.24	4.70 ± 0.160	0.36 ± 0.04
Potassium (mg L^−1^)	0.73 ± 0.03	0.04 ± 0.004	3.43 ± 0.04
Sodium (mg L^−1^)	0.30 ± 0.01	0.04 ± 0.002	60.70 ± 0.47

### Energy and economic aspects of producing Mg-laden microalgal biochar through ECF for seeding struvite crystallization

Microalgal cultivation did not require any major energy or cost inputs (wastewater grown) other than occasional aeration to suspend the culture. The overall energy consumed for integrated harvesting and modification of microalgae was calculated to be 4.03 kWh kg^−1^, which was found to be less than other conventional recovery methods (Supplementary Table S1). In this study, the main cost governing factor of using magnesium electrode corresponds to its material cost. However, compared to the chemical cost of MgCl_2_.6H_2_O (HiMedia), the magnesium anode cost is negligible. For pre-treating 1 kg of microalgal biomass, the cost incurred by using MgCl_2_.6H_2_O and Mg anode is 222 USD and 3.20 USD, respectively, as per the results of this study. The total electrical operating cost for the optimized integrated ECF technique aimed at pre-treatment of algal biomass is 2.22 USD kg^−1^. The energy consumed for drying and pyrolysis of Mg-laden microalgal biomass in this work was calculated and found to be 0.50 and 2.60 kWh kg^−1^ biomass, respectively. Here, the operational costs only include electricity charges as the process did not any gas sparging corresponding to 0.12 USD kg^−1^. For struvite crystallization, a Jar test apparatus was used for mixing Mg-MiB in synthetic urine for possible struvite precipitation and later the SMB composite obtained was dried using a hot air oven. The operational cost for this unit process was calculated to be 0.14 USD kg^−1^. The overall operating cost of this integrated process is 2.48 USD kg^−1^ with a total EC of 10.76 kWh kg^−1^.

## Discussion

The microalgal concentration yielded in this study can be considered as optimum for harvesting algae through flocculation as reported in the literature^15^. The surface properties of microalgae during growth play a crucial role in harvesting and hence study of factors such as biomass concentration and microalgal growth phase becomes essential.

The electrochemical system offers cations and gas bubbles necessary for both processes to take place with the help of a polyvalent metal anode and an inert cathode. In case of magnesium sacrificial anode, magnesium dissolves in the form of Mg^+^ ions. Later, the dissipation is assisted by hydrogen gas emanation. As pH rises due to OH^−^ ions dissolution, Mg(OH)_2_ is formed on the surface of microalgal cells and anode^11^.

Current density plays a significant role in determining the dissolution rate of ions from the anode. High current input fastens the ion dissipation and thus algal removal, within a short electrolysis time. However, CD also corresponds to increase in the overall cost of the algal products^16^. It is reported that the energy consumed with higher CD at short time is much higher than consumed with lower CD at longer time^15^. At lower CD the gas bubbles get attached to the cathode and decreases the flotation efficiency^17^. Also, high and rapid CD input has a relatively lesser impact on algal pigments as stated in literatures^15,16^. Hence, optimization of CD is essential and the CD 40.78 mA cm^−2^ used in this study is in correspondence with literature for recovery of algal strains such as *Scenedesmus quadricauda, Chlorococcum sp.* and *Tetraselmis sp.*^18,19^. At such CD, the bubbles detach easily from the cathode due to smaller size and facilitate better flotation^17^.

Results similar to the IED optimized in this work were observed by Wiley & Trent^20^, where aluminium and titanium/iridium oxide electrodes were used to harvest 99.5% of *Chlorella vulgaris* in an ECF system. Also, the trend observed in Fig. 1b could be because of the electric conductance decreases with increase in distance. As an outcome of this, the power consumption increases. As the interelectrode gap increases, the ohmic loses are higher leading to resistance in mass transfer and charge transfer kinetics. Another possible explanation can be the decrease in faradaic yield with increase in IED, resulting in reduction of ion release^15,21^.

The pH rise towards alkalinity is due to the release of hydroxyl ions at the cathode and this buffering effect is an added advantage of ECF. In case of magnesium electrodes, Mg^+^ ions are released at initial stages, followed by hydrogen gas release. The formation of Mg(OH)_2_ on the surface of anode can prevent further corrosion, resulting in decrease of ion impregnation on microalgal cell surface. According to Pourbaix diagram, Mg corrodes as Mg^2+^ ions and remains in the same ionic state up to pH 13. The corrosion increases at higher potentials; however, Mg(OH)_2_ layer stabilizes only at pH greater than 11^22^. These explanations elucidate that the electrode material and pH conditions maintained in this study is suitable for efficient microalgal harvesting and pre-treatment.

Conductivity, salinity and TDS are interrelated characteristics which have analogous effect on microalgal harvest. Higher salinity of the electrolyte increases conductivity and thus ion release required for recovery. In this study, the electrical conductivity and salinity values are lower compared to other studies. This could be because the microalgal consortium was obtained from freshwater lake and acclimatized in diluted urine, as the strains were not able to tolerate high salinity of concentrated urine. Also, no salt supplementation was provided to the media as the residual supernatant was aimed to be used for further algal cultivation. Such lower ionic strength solutions also offer benefits of smaller and stable bubble formation for efficient bubble-cell adhesion and flotation^17^. The slight increase in conductivity, salinity, and TDS of the medium after harvesting could be because of the residual magnesium ions dissipated into the medium as a result of electro-coagulating microalgal cells using magnesium anode. The composition of this algal growth media is shown in Table 2. Both electrical conductivity and salinity have an inverse effect on the energy consumption of the system. In most of the studies, NaCl is added in large quantities to improve the salinity and alleviate energy requirement. However, this will increase the cost and pose several drawbacks associated with utilization of media for consecutive algal growth with biological effect of NaCl hindering the mobility of microalgae. In addition, the change in double layer compression of algal cells during ECF can be induced by salinity of the medium, assisting microalgal aggregation^15^.

The ion content incorporated with biochar is much higher in this study (28.19 wt% Mg) compared to other literatures. Chabi et al*.*^9^ reported 7.17 wt% aluminium and 16.67 wt% boron incorporation in *Chlorella sp.* using electrocoagulation as a pre-treatment strategy for tetracycline removal. Thant et al.^6^ conducted chemical impregnation which led to increase in magnesium content up to 11.05 wt%. It is also noteworthy that the chlorine concentration is minimum in electro-modified Mg-MiB. Though use of MgCl_2_ is proven to be effective for chemical pre-treatment, it’s use not only contributes to magnesium but also to chlorine ions. In fact, the chlorine concentration of biochar was very high compared to magnesium, in chemical treatment. The remains of chlorine ions can lead to various environmental problems. Their presence in the aqueous medium, can cause oxidation into Cl_2_ and further into HClO, which are strong oxidizing agents that might be detrimental to the microalgal cells^15^. This might hinder the reuse of the residual supernatant after struvite precipitation for further algal cultivation. Also, electro-modification of raw biomass offers physicochemical benefits to biochar by enhancing the crystallinity, surface area and impregnation of ion of the surface. The comparison of various studies carried out on electro- and chemical modification are shown in Table 3 and confirms that ECF pre-treatment strategy is time saving, simple and efficient, and hence can be considered as the ideal method for engineering biochar.

**Table 3:** Comparison of pre-treatment duration and ion impregnation capacity between electro- and chemical modification techniques and their intended applications.

Biochar pre-treatment technique	Feedstock	Electrode material/chemical	Preparation time (min)	Application	References
Electro-modification	Microalgal consortium	Magnesium	20	Mg source and seed for struvite crystallization	This work
	Brown marine macroalgae	Graphite; MgCl_2_ electrolyte	10	Phosphate removal	^23^
	*Laminaria japonica*	Aluminium	5	Phosphate adsorption	^24^
	*Laminaria japonica*	Aluminium; MgCl_2_ electrolyte	4.8	Phosphate adsorption	^10^
	*Chlorella sp.* (PTCC 6010)	Aluminium-Boron	17.65	Tetracycline removal	^9^
Chemical modification	Microalgal consortium	MgCl_2_.6H_2_O	480	Mg source and seed for struvite crystallization	This work
	Sewage sludge waste and food waste	MgCl_2_.6H_2_O	480	Phosphate adsorption as struvite	^6^
	Corncob	MgCl_2_ and CaCl_2_ solution	360	Phosphate recovery	^25^
	Cypress sawdust	MgCl_2_ solution	180	Phosphate recovery	^26^
	Taro straw, corn straw, cassava straw, Chinese fir straw, banana straw, *Camellia oleifera* shell	MgCl_2_	240	Nitrogen and phosphorus adsorption	^13^

The insignificant variation of ammonium during struvite crystallization could be because of loss as ammonia during the experiments^27^. The observations of the control experiment are in corroboration with PO_4_^3−^ recovery results obtained by Xu et al*.*^28^, during struvite precipitation from urine. Though the PO_4_^3−^ recovery in positive control was quite high, most of struvite fines were found floating over the supernatant or stuck to the walls of the experimental apparatus and hence were hard to recover. This yield is considerably high as the medium was finally centrifuged to recover all the crystals. However, at a bench or pilot scale level, this process could be energy-intensive.

**Figure 5:**
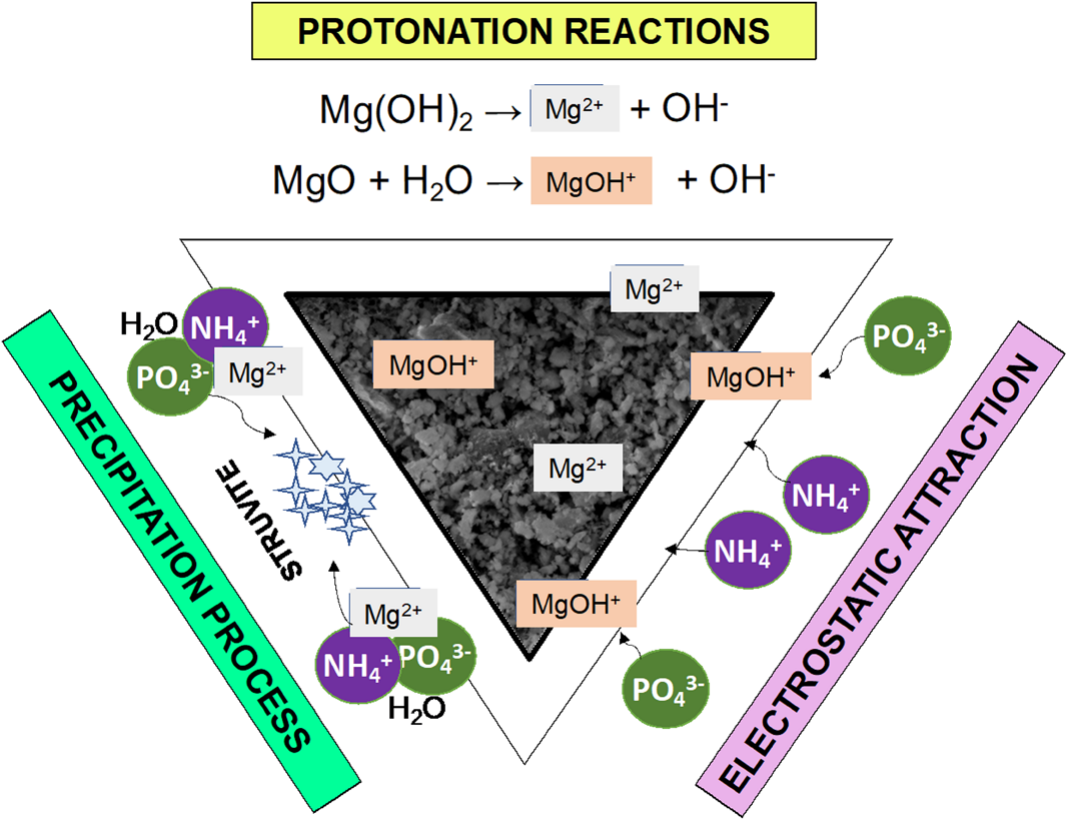
Possible mechanisms for struvite formation and deposition on Mg-laden microalgal biochar seeds.

Synthetic struvite crystals obtained in the crystallization experiments had distinct rod-shaped crystals as mentioned in the literature^29^. However, the SMB composites had flower-like or star-like crystals which can be attributed to the adsorption of phosphate and ammonium ion on seeding sites containing impregnated magnesium nanoflakes (Supplementary Fig. S4). Similar structures have also been reported in previous studies by Lee et al*.*^30^. The crystal formation qualifies its application as a soil amendment that can supplement nutrients to the plant directly and indirectly. The mechanisms behind this structure formation are shown in Fig. 5. The mechanisms were combined based on the evidences of the qualitative characterizations performed and postulations provided in earlier studies^14,31^. It can be observed from FTIR analysis that the transmission of PO_4_^3−^ in SMB composite is lower than synthetic struvite indicating presence of higher number of bonds, which also supports SEM–EDS results in terms of higher recovery. The functional groups in SMB composite are more diverse as it consists of several nutrients. The comparison of nutrition composition between microalgal biochar, synthetic struvite and SMB composite is shown in Supplementary Table S2. It can be seen that all the inorganic elements such as phosphorus, magnesium, potassium, calcium and sodium are comparatively higher in SMB composite. These evidences support the experimental data and FTIR analysis of enhanced phosphate recovery as struvite in terms of quantity and quality.

It can be understood from the microalgal harvesting and struvite precipitation experiments that almost 98% of the total media volume is left over after the processes. This medium is either discarded or the end use if left undiscussed in previous works. In order to reduce the water footprint and maintain sustainability in the experiments conducted, the residual supernatants after each unit process were characterized to evaluate its suitability for successive cultivation of microalgae. Considering that microalgal growth takes place in a neutral to alkaline pH, the characteristics of the supernatants should be adequate^32^. The results show that a combined use of both the supernatants will provide all the necessary nutrients in optimum level for microalgal growth, except phosphorus. Hence, for consecutive algal cultivation, external addition of phosphorus can be facilitated or if the culture medium is wastewater as in this study, the residual supernatants can be utilized as dilution mediums, which can reduce fresh water use. The algae cultivated can further modified and used for the production of microalgal biochar.

The energy consumption is less due the advantage of quick anodic dissolution magnesium. In addition to this, non-galvanic corrosion of magnesium sacrificial electrode is an added advantage with respect to microalgal recovery and energy consumption^33^. Compared to other commonly used metals such as aluminium or iron, the quasi-hydroxide layers formed on the anode prevents further corrosion or further dissipation of ions required for electro-modification. This layer formed on magnesium sacrificial anode is much unstable or even slightly soluble^22^. Hence, magnesium offers poor resistance to pitting and is a suitable material for electrical pre-treatment of biochar with minimum energy requirements.

Though MgCl_2_.6H_2_O is regarded as the most expensive magnesium salt, it is recommended because of its high impregnation efficiency^33^ and generally, a higher concentration solution is used initially and later most of it is lost during washing of biochar, which is not necessary for electro-modified chars. Also, pure magnesium electrode is used in this study, whose cost is at least 10 times less than the commonly used AZ31 Mg alloy, without compromising the intended efficiency^34^. The operating cost of ECF process much lower than the costs reported by Pirwitz et al*.*^35^ for electroflocculation (21.95 USD kg^−1^), chemical flocculation (Alum: 20.17 USD kg^−1^) and centrifugation (217.13 USD kg^−1^) of *Dunaliella salina* (21.95 USD kg^−1^) using aluminium electrodes. However, these costs also include manpower and tax, which are neglected in this study. It is reported that struvite seeding requires significant mixing to maintain the seeds in suspension or high flowrate during recirculation to ensure formation of agglomerates which might add up to the overall energy and cost^36^. In spite of this, the combined EC is comparable or lower than single unit process of ECF or centrifugation for microalgal harvesting as shown in Supplementary Table S1.

In this work, the microalgae were cultivated using diluted urine, a cheap but nutritious media, under natural conditions of light, photoperiod, and atmospheric CO_2_. These circumstances can reduce the cost of the biomass feedstock by substituting the conventional growth media and artificial illumination apparatus. The culture was then simultaneously harvested and pre-treated using a sacrificial magnesium anode and inert carbon cathode in an integrated electrocoagulation-flotation system. This technique was not only efficient in harvesting and magnesium impregnation but also in terms of energy consumption and cost compared to other conventional techniques. The microalgae collected was pyrolyzed at a high temperature to produce Mg-laden microalgal biomass which in turn was used as a seed material in struvite crystallization to enhance the recovery of phosphorus and ammonium from urine. In addition to consecutive utilization of products generated in each unit process, the excess supernatants left out after microalgal harvesting and struvite crystallization were assessed to determine the suitability of physicochemical conditions for further microalgal cultivation. It was found that a blend of the supernatants could not only satisfy the nutrient requirement but also reduce the water usage necessary for dilution of urine. The microalgae cultivated using these supernatants can again be involved in the process of microalgal biochar production and thereby for seeding struvite crystallization. It is mention-worthy that the cumulative energy consumed by all the unit processes was lower than some of the conventional microalgal harvesting techniques. Also, the cost incurred by this regenerative economic system was significantly lower due to the use and reuse of cheap nutrient media, single-step integrated technique to efficiently harvest and pre-treat biomass and usage of Mg-laden microalgal biochar as a seed material and source of magnesium for struvite recovery. Kholssi et al.^44^ has stressed on the various opportunities presented by microalgae for achieving sustainable development in a such circular bioeconomy. These prospects include high value-added products such as pigments, polyunsaturated fatty acids, bioactive compounds, and valorization of the biomass itself as fertilizer or for production of biochar. Such concepts have also been explored for treating anaerobic digestate using microalgae and later using them in the energy sector or animal feed^45^. Extraction of bio-oil from algae prior to pyrolysis was also investigated for possible application in a circular economy cycle and this strategy resulted in an increased biochar production by 25–33%^46^. Microalgae are generally attributed to the context of circular bioeconomy due to their ability to rejuvenate, attenuate nutrient and organic load in wastewater, alleviate greenhouse gas emissions while contributing to food security. Hence, microalgae can be certainly incorporated in a circular process of wastewater remediation, zero carbon emission and further be used as a feedstock in several other systems to kindle sustainable development^45^.

## Conclusion

Experimental and characterization results revealed that electro-modification of microalgae with ECF was more efficient than chemical with respect to Mg-impregnation and cost. Seeding struvite crystallization with engineered microalgal biochar enhanced phosphorus recovery (> 10–15%) and crystal size (~ 2 times). The nutritional composition of SMB composite justifies the hypothesis of this study of achieving a self-sustainable, organic and direct-use fertilizer containing all the major and minor essential nutrients required by plants. Hence, integration of ECF-harvesting and surface modification, pyrolysis, and struvite precipitation is a sustainable strategy in terms of energy, cost, waste management and nutrient recovery, paving way for a circular bioeconomy.

## Methods

### Microalgal cultivation

Microalgal consortium samples were collected from Naga Pond at National Institute of Technology (NIT) Rourkela, India (22.2604° N, 84.8536° E) and were acclimatized for a period of 3 months in an optimized urine concentration of 6.5%. The dominant species present in the consortium were *Chlorella sp., Spirulina sp.* and *Scenedesmus sp.,* and *Synechocystis sp.* These samples were cultivated in a closed photobioreactor with 50 L working volume and natural conditions of solar radiation (0–900 W m^−2^), temperature (25 ± 5 °C) and photoperiod (12:12) until the desired concentration for harvesting is attained. The culture was occasionally aerated to enhance the growth of cells in suspension. Microalgal growth and productivity was monitored on set intervals of 72 h to determine the suitable harvest period.

### Simultaneous microalgal harvesting and pre-treatment using integrated electrocoagulation-flotation (ECF)

Once the microalgal growth attained stationary phase, it was harvested using an integrated electrocoagulation-flotation technique. An aspirator bottle (1 L) with stopcock was used as the apparatus for experiments for ease of microalgal recovery and residual supernatant decantation. Magnesium and carbon electrodes were used as sacrificial anode and inert cathode, respectively, for simultaneous release of Mg^2+^ and gas bubbles. The current (0–2 A) and voltage (0–30 V) were supplied to the electrodes through a multiple DC power supply (Mars Me-230). The total surface area (TSA) of carbon (TSA_carbon_ = 41 cm^2^) was fixed to be slightly higher than magnesium (TSA_magnesium_ = 38 cm^2^) to enhance flotation as and when the microalgal flocs were formed. The medium was subjected to fast mixing (100 rpm) followed by slow stirring (60 rpm) using a magnetic stirrer.

The parameters that significantly influence the process of ECF such as current/current density, voltage, reaction time, inter-electrode distance and magnesium dosage was optimized with respect to algal recovery and energy consumption. The interelectrode distance between the electrodes was maintained using inert spacers. The current density (CD) was calculated using Eq. (1)^37^.

1 $$CD=\frac{I}{A}$$

where, $I$ and $A$ are current supplied (A) and effective surface area of the electrode (cm^2^), respectively.

During ECF, the samples were collected at intervals of 5 min until the supernatant became completely devoid of microalgal cells and measured for optical density at 680 nm using a Go Direct® SpectroVis® Plus Spectrophotometer (Vernier, USA) to monitor the recovery. All the experiments were carried out in triplicates and the standard deviations were noted. The microalgal harvesting efficiency (MHE) and energy consumed during the ECF process was calculated using Eq. (2)^32^ and (3)^16^, respectively.

2 $$MHE=\frac{{A}_{I}-{A}_{F}}{{A}_{I}}$$

where, ${A}_{I}$ and ${A}_{F}$ are initial and final absorbance of microalgal sample at 680 nm, respectively.

3 $$Energy\, consumption\, (kWh {kg}^{-1})=\frac{v.I.t}{1000\times V\times MHE\times {C}_{i}}$$

where, $v$ is the voltage (V), $I$ is the current (A), $t$ is the ECF process time (h) and $V$ is the volume of the microalgal sample (m^3^), $MHE$ is the microalgal harvesting efficiency and ${C}_{i}$ is the initial microalgal concentration (kg m^−3^).

The variations of physicochemical properties such as pH, conductivity, salinity and TDS in the microalgal medium before and after harvesting were monitored using a multiparameter water quality analyser (Model no. LMMP-30, Labman Scientific Instruments Pvt. Ltd., India). The amount of magnesium dissipated from the anode was measured as the difference between the weight of anode before and after the experiments. The residual magnesium concentration in the medium was colorimetrically determined using magnesium reagents from Hanna Instruments (HI937520-03) with a double beam UV–Vis Spectrophotometer (Model 2230, Systronics, India) at 466 nm.

For comprehending the pre-treatment efficacy of ECF, a comparative study between chemical and electro-modification of microalgal biomass was conducted. The preparation method for chemical impregnation of Mg^2+^ ions was adapted from Thant et al.^6^ and MgCl_2_.6H_2_O procured from Hi-Media was used as the source of ions.

### Production of Mg-laden microalgal biochar through pyrolysis

The Mg-laden microalgal flocs floating on the surface of the apparatus were collected and dried at 80 °C. The dried microalgae were then loaded into a crucible with less than 10% headspace and sealed completely with aluminium foil for maintaining an oxygen limited environment. The magnesium pre-treated algal biomass was then subjected to thermochemical conversion through pyrolysis at a temperature of 480 °C and heating rate of 5 °C min^−1^ in a top loading muffle furnace for 45 min. After pyrolysis, the samples were allowed to cool down to room temperature and labelled as Mg-laden microalgal biochar (Mg-MiB). The yield of biochar was calculated using Eq. (4)^38^.

4 $$Yield\, of\, Mg-MiB\, \left(\%\right)=\frac{{M}_{Mg-MiB}}{{M}_{Mg-microalgae}}\times 100$$

where, ${M}_{Mg-MiB}$ and ${M}_{Mg-microalgae}$ are the mass of Mg-laden microalgal biochar (g) and Mg-laden microalgal biomass (g), respectively.

### Struvite crystallization by seeding with Mg-laden microalgal biochar

The nutrient medium used for recovery of struvite is synthetic urine prepared as per the composition reported in Liao et al*.*^39^ (2.30 g L^−1^ Na_2_SO_4_; 2.10 g L^−1^ NaH_2_PO_4_; 3.60 g L^−1^ NaCl; 4.20 g L^−1^ KCl; 9.60 g L^−1^ NH_4_Ac; 17.04 g L^−1^ NH_4_Cl; 6.89 g L^−1^ NaOH; 21.40 g L^−1^ NH_4_HCO_3_) and the pH was maintained at 9.01 ± 0.2. In this study, the magnesium required for struvite crystallization was supplied through Mg-MiB. The concentration of magnesium was optimized by varying the dosage of Mg-MiB (0, 0.50, 1.00, 1.50, 2.00, 2.50 and 3.00 g L^−1^) with respect to PO_4_^3−^ and NH_4_^+^ recovery, and the resultant Mg^2+^ concentration in the medium and yield of struvite-microalgal biochar (SMB) composite. For positive control, MgCl_2_.6H_2_O was used to precipitate struvite to determine PO_4_^3−^ recovery and crystal size. Optimization experiments were carried out by subjecting the reaction mixtures to stirring at 150 rpm for 10 min in a Jar Test Flocculator, followed by a retention time of 8 h. The residual supernatant was stored for further analysis and the struvite residue at the bottom was dried at 40 °C overnight and kept for quantitative and qualitative characterizations. The PO_4_^3−^concentration was estimated by Murphy and Riley stannous chloride method with absorbance at 650 nm using a UV–Vis Spectrophotometer (Model 2230, Systronics, India). Phenol-hypochlorite spectrophotometric method was used for the determination of NH_4_^+^ concentration at 630 nm^1^. Mg^2+^ concentration was measured using the Standard methods for the examination of water and wastewater, 18th edition, Calmagite method (HI-937520–03, Hanna reagents, India). All the experiments were carried out in triplicates.

The calcium and nitrate concentrations in the supernatant were measured using respective ion-selective electrodes from Vernier, USA. Sodium and potassium concentrations were estimated using a flame photometer (Model no. 1385, ESICO, India). All the experiments were carried out in triplicates and the standard deviations were recorded. The PO_4_^3−^ and NH_4_^+^ recovery was calculated using Eqs. (5) and (6), respectively^1^. The struvite yield (g L^−1^) was the difference between SMB composite yield and Mg-MiB dose. Struvite produced from a synthetic urine medium using commercial MgCl_2_.6H_2_O (HiMedia, India) was considered as the positive control and sample without biochar as the negative control.

5 $${PO}_{4}^{3- }\mathrm{recovery}=\frac{{{PO}_{4}^{3- }}_{I}-{{PO}_{4}^{3- }}_{F}}{{{PO}_{4}^{3- }}_{I}}\times 100 (\mathrm{\%})$$

where, ${{PO}_{4}^{3- }}_{I}$ and ${{PO}_{4}^{3- }}_{F}$ are concentrations of phosphate (mg L^−1^) before and after struvite precipitation, respectively.

6 $${NH}_{4}^{+ }\mathrm{recovery}=\frac{{{NH}_{4}^{+ }}_{I}-{{NH}_{4}^{+ }}_{F}}{{{NH}_{4}^{+ }}_{I}}\times 100 (\mathrm{\%})$$

where, ${{NH}_{4}^{+ }}_{I}$ and ${{NH}_{4}^{+ }}_{F}$ are concentrations of ammonium (mg L^−1^) before and after struvite precipitation, respectively.

### Analysis of Mg-laden microalgal biochar and struvite-microalgal biochar composite characteristics

The Mg-laden microalgal biochar was characterized using Fourier Transform Infrared (FTIR) Spectroscopy (MN344, Alpha ATR-FTIR, Bruker, Germany) for analysing the functional groups present. X-Ray Diffraction (XRD) analysis was carried out to study the crystalline phases of Mg-MiB with a diffractometer equipped with Cobalt-Iron radiation (AXS D8, Bruker, Germany). The surface morphology and chemical composition was elucidated using Scanning Electron Microscopy—Energy Dispersive X-ray spectroscopy (SEM–EDS) (JEOL JSM- 6480 LV, Oxford Instruments, UK). Once the struvite crystallization experiments were carried out, the quality and composition of the crystals were analysed using FTIR and SEM–EDS, respectively. The variation in particle size of struvite crystals with seeding was examined using a particle size analyzer (Mastersizer 2000, Nano ZS, Malvern, U.K.).

### Energy and cost evaluation

The operating cost of the ECF system was calculated using Eq. (7)^34^.

7 $$EOC=EC\times EEP+UEMD\times EMP$$

where, $EOC$ is the electrical operating cost for harvesting microalgae (USD kg^−1^), $EC$ is the energy consumed by the ECF process (kWh kg^−1^) as per Eq. (3), $EEP$ is the electrical energy price (Rs. 3 kWh^−1^ (Government of Odisha tariff) equivalent to 0.04 USD kWh^−1^), $UEMD$ is the unit electrode material demand (2/3 for magnesium) and $EMP$ is the electrode material price (3.31 USD kg^−1^). For other unit processes, the energy consumption was calculated as kWh based on the current, voltage, processing time and operating conditions.

## Supplementary Information

Supplementary Information.

## Data Availability

All data generated or analyzed during this study are included in this article and its supplementary information file.
