# Supplementary material for: Integrated electrocoagulation-flotation of microalgae to produce Mg-laden microalgal biochar for seeding struvite crystallization
Source: Sci Rep. 2022 Jul 6;12:11463. doi: 10.1038/s41598-022-15527-6 (PMC9259614; doi:10.1038/s41598-022-15527-6)
Supplement: Supplementary file 1 — Supplementary Information. [file 41598_2022_15527_MOESM1_ESM.docx]

**Integrated electrocoagulation-flotation of microalgae for producing Mg-laden microalgal biochar for seeding struvite crystallization**

*Krishnamoorthy Nageshwari ^1^, Scott X. Chang ^2^, Paramasivan Balasubramanian ^1,^ **

^1^ Department of Biotechnology & Medical Engineering, National Institute of Technology Rourkela, Odisha, India – 769008

^2^ Department of Renewable Resources, University of Alberta, Edmonton, Alberta T6G 2E3, Canada

* Corresponding author: biobala@nitrkl.ac.in; Tel: (+91) 661 246 2297 [**http://orcid.org/0000-0002-3821-5029**](http://orcid.org/0000-0002-3821-5029)

**18 May 2022**


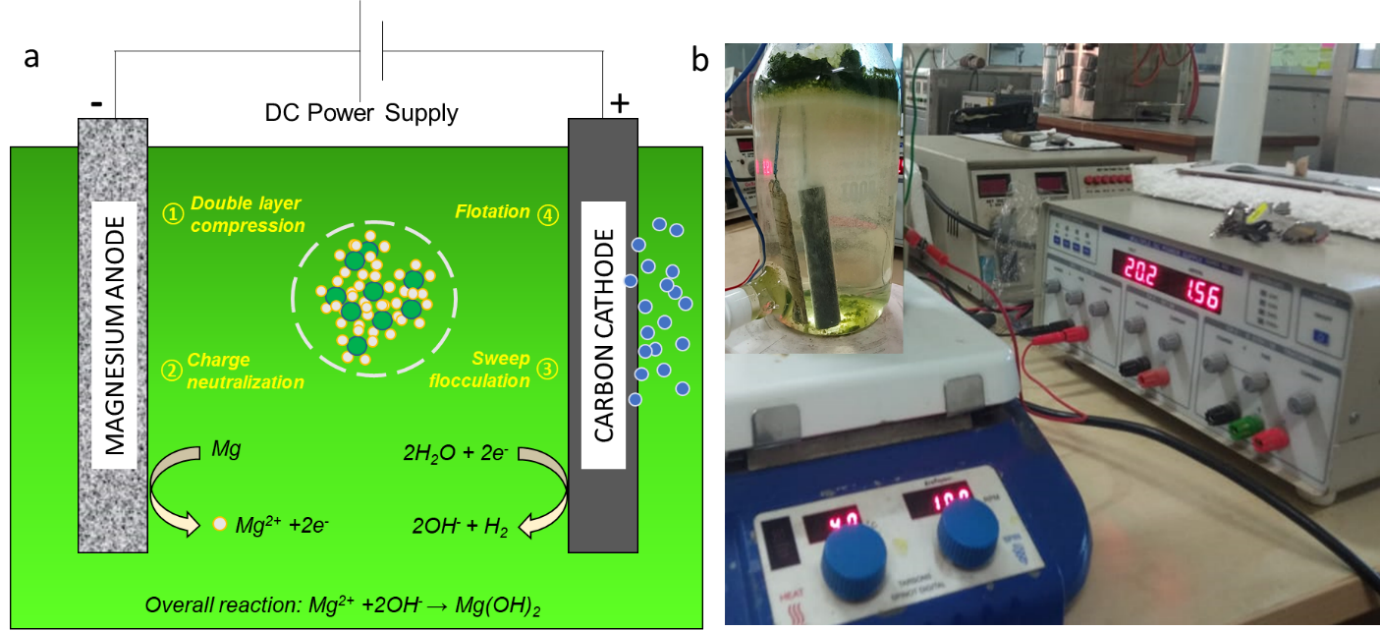


**Fig. S1**: a) Schematic representation of Electro-coagulation-floatation mechanisms with Mg-C electrodes; b) Electro-coagulation-floatation experimental set-up at laboratory scale

**Fig. S2**: Variation in the physicochemical parameters such as a) pH, b) electrical conductivity, c) total dissolved solids and d) salinity of microalgal medium before and after harvesting


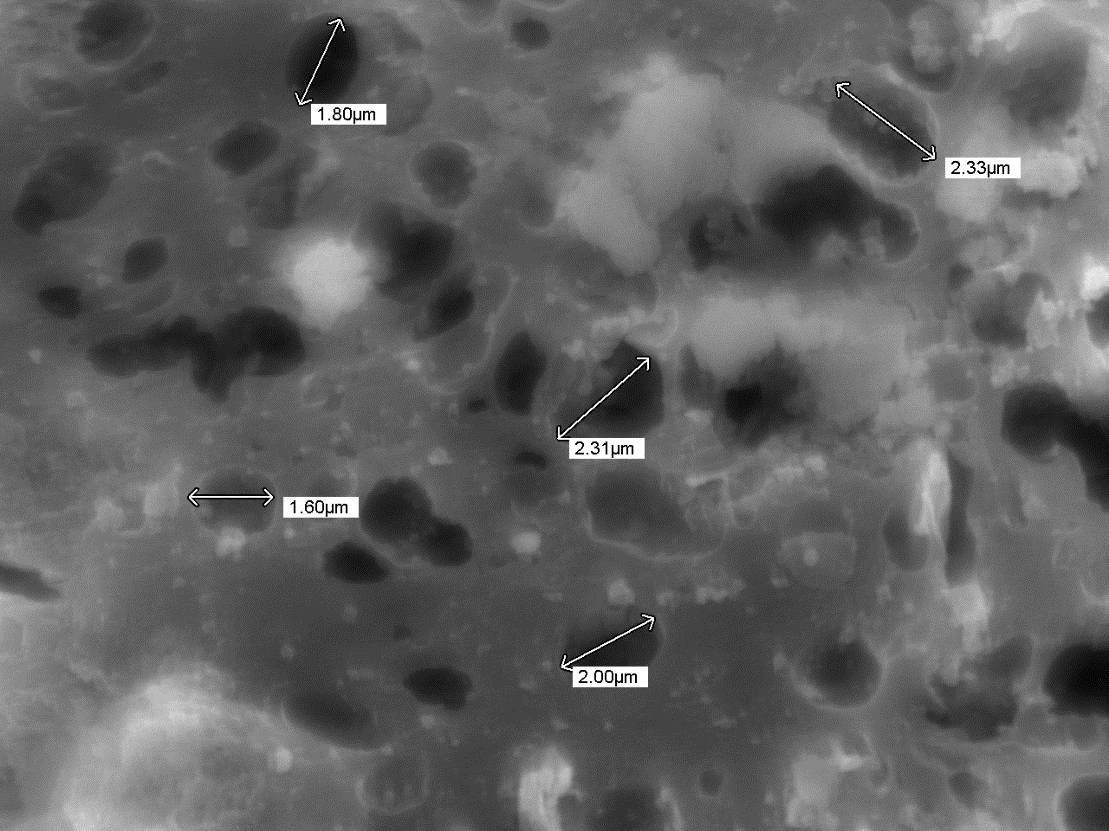


**Fig. S3**: Analysis of Mg-laden microalgal biochar pore size using scanning electron microscopy


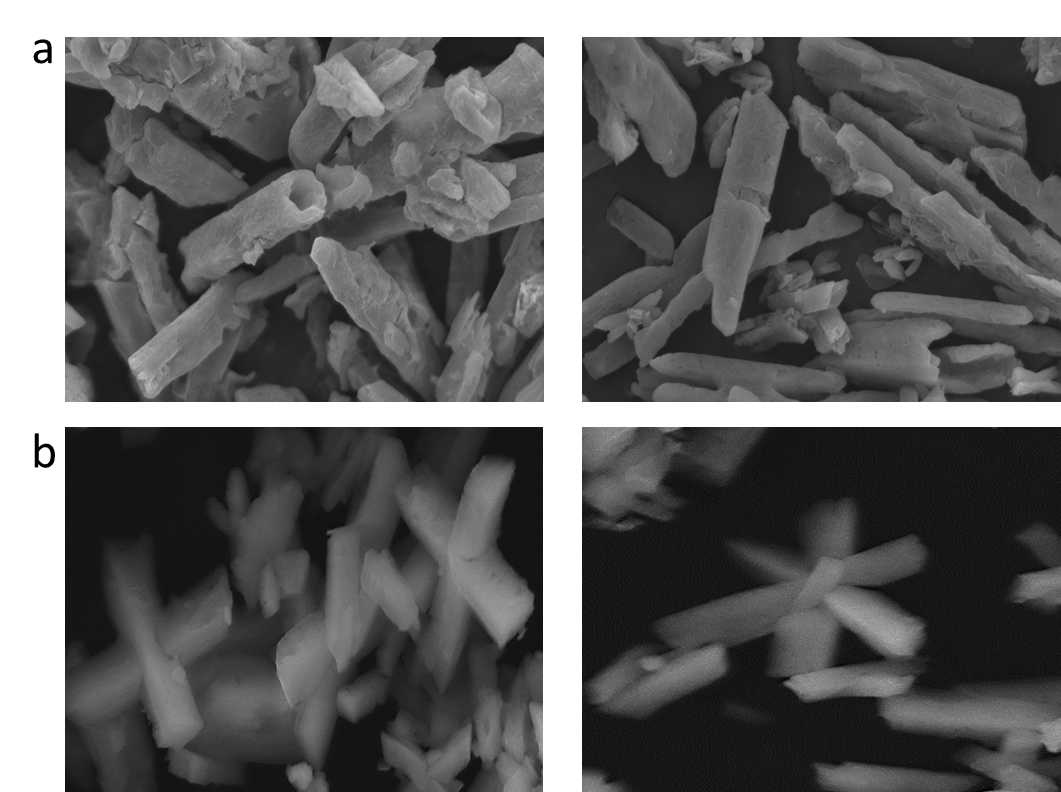


**Fig. S4**: Scanning electron microscopy images of a) synthetic struvite crystals b) struvite-microalgal biochar composite

**Fig. S5:** Scanning Electron Microscopy-Energy Dispersive X-ray spectroscopy analysis of microalgal biochar ^7^

**Table S1**: Comparison of energy consumption between electro-coagulation-flotation and other methods of microalgal harvesting

| Microalgal harvesting technique | Microalgal species | Electrode/chemical used | Time taken (min) | Efficiency (%) | Energy consumed (kWh kg^-1^) | References |
| --- | --- | --- | --- | --- | --- | --- |
| Electro-coagulation-flotation | Microalgal consortium | Magnesium | 20 | 98.0±0.4 | 4.03 | This work |
| Electrocoagulation | *Tetraselmis sp.* | Aluminium | 10 | 90.9 | 4.62 | ^1^ |
| Chemical & electrocoagulation | *Scenedesmus sp.* | Aluminium sulphate & graphite |  | >90 | 4.04 | ^2^ |
| Electro-coagulation-flocculation | *Chlorella vulgaris* | Aluminium | 60 | 99 | 9.4 | ^3^ |
| Centrifugation | *Ankistrodesmus*  *Falcatus* | - | 20 | 93.4± 0.35 | 65.3 | ^4^ |
| Chemical flocculation | *Ankistrodesmus*  *Falcatus* | Alum | 60 | 86.1 ± 0.09 |  | ^4^ |
| Centrifugation | *Chlorella vulgaris* | - | - | - | 16 | ^5^ |
| Biological flocculation | *Scenedesmus obliquus* | Chitosan | 60 | 77.0 ± 0.5 | - | ^4^ |
| Electro-coagulation-flocculation | *Tetraselmis sp.* | Stainless steel (Iron) | 15 | 99 | 9.16 | ^6^ |
| Electro-coagulation-flocculation | *Chlorococcum sp.* | Stainless steel (Iron) | 15 | 98 | 4.44 | ^6^ |

**Table S2**: Nutrient composition (%) of struvite-microalgal biochar composite with comparison to synthetic struvite and microalgal biochar

| Nutrient element | Synthetic struvite | Microalgal biochar | Struvite-microalgal biochar composite |
| --- | --- | --- | --- |
| Phosphorus | 11.7 | 0.5 | 23.0 |
| Magnesium | 10.1 | 0.7 | 15.0 |
| Carbon | 8.3 | 40.7 | 18.3 |
| Oxygen | 48.5 | 34.3 | 37.6 |
| Nitrogen | 3.9 | - | 5.4 |
| Potassium | 9.0 | 0.3 | 0.3 |
| Calcium | 0.4 | 1.9 | 0.3 |
| Sodium | 0.6 | - | 0.5 |

**References**

1. Hawari, A. H., Alkhatib, A. M., Das, P., Thaher, M. & Benamor, A. Effect of the induced dielectrophoretic force on harvesting of marine microalgae (*Tetraselmis sp.*) in electrocoagulation. *J. Environ. Manage.* **260**, 110106 (2020).

2. Liu, S., Abu Hajar, H. A., Riefler, G. & Stuart, B. J. Investigation of electrolytic flocculation for microalga: *Scenedesmus sp.* using aluminum and graphite electrodes. *RSC Adv.* **8**, 38808–38817 (2018).

3. Fayad, N., Yehya, T., Audonnet, F. & Vial, C. Harvesting of microalgae *Chlorella vulgaris* using electro-coagulation-flocculation in the batch mode. *Algal Res.* **25**, 1–11 (2017).

4. Guldhe, A., Misra, R., Singh, P., Rawat, I. & Bux, F. An innovative electrochemical process to alleviate the challenges for harvesting of small size microalgae by using non-sacrificial carbon electrodes. *Algal Res.* **19**, 292–298 (2016).

5. Vandamme, D. *et al.* Evaluation of electro-coagulation-flocculation for harvesting marine and freshwater microalgae. *Biotechnol. Bioeng.* **108**, 2320–2329 (2011).

6. Uduman, N., Bourniquel, V., Danquah, M. K. & Hoadley, A. F. A. A parametric study of electrocoagulation as a recovery process of marine microalgae for biodiesel production. *Chem. Eng. J.* **174**, 249–257 (2011).

7. Pathy, A., Krishnamoorthy, N., Chang, S. X. & Paramasivan, B. Malachite green removal using algal biochar and its composites with kombucha SCOBY: An integrated biosorption and phycoremediation approach. *Surfaces and Interfaces* **30**, 101880. https://doi.org/10.1016/j.surfin.2022.101880 (2022).
